# Scoping review of contemporary adult venoarterial extracorporeal membrane oxygenation (VA ECMO) selection criteria and decision-making mechanisms

**DOI:** 10.1051/ject/2026009

**Published:** 2026-06-19

**Authors:** Amrita K Johal

**Affiliations:** 1 Kelowna General Hospital Kelowna Canada

**Keywords:** Venoarterial extracorporeal membrane oxygenation, ECMO team, ELSO, ECMO patient selection, SAVE score, Modified SAVE score

## Abstract

Adult venoarterial extracorporeal membrane oxygenation (VA ECMO) is a costly life support therapy, where patient selection remains a significant hinge-point in determining patient outcomes. Despite this, few sites have formalized patient selection criteria, and even fewer have a robust multidisciplinary ECMO team to determine patient candidacy. Analyzing current literature revealed weaknesses in existing research on VA ECMO patient selection criteria. Significant issues include the use of small sample sizes, lack of randomized controlled trials, lack of personalization of ECMO initiation decisions, study heterogeneity, and inclusion of patients who received ECMO without including patients who were considered for but not given ECMO. Among the available studies, the Survival After Venoarterial ECMO (SAVE) score has shown the greatest promise in selecting VA ECMO patients, excluding those undergoing extracorporeal cardiopulmonary resuscitation (ECPR) and postcardiotomy (PC) ECMO. The SAVE score has demonstrated good discrimination in non-ECPR and non-PC ECMO groups. Further research is needed on the predictive value of SAVE score risk classes and the discrimination of the SAVE score excluding PC ECMO and ECPR groups. The modified SAVE score, which also includes lactate assessment, has shown the greatest promise for selecting ECPR and PC ECMO patients for VA ECMO. The modified SAVE score requires more external validation. Site-level research indicates that consultation with a multidisciplinary ECMO team, which collectively makes decisions about ECMO candidacy, has resulted in significantly improved patient survival outcomes compared with a one- or two-physician decision-making mechanism for VA ECMO initiation. The ECMO team approach is a rich area for future research, and sites should publish their patient outcomes before and after implementation.

## Introduction

In venoarterial extracorporeal membrane oxygenation (VA ECMO), robust selection criteria identifying adult candidates who benefit are lacking [1]. All types of ECMO, including extracorporeal cardiopulmonary resuscitation (ECPR), VA, and venovenous (VV) ECMO, have been considered a last-resort salvage therapy for patients in critical condition as a bridge to heart and/or lung recovery, bridge to heart and/or lung transplant, or bridge to a long-term therapy such as Ventricular Assist Device (VAD) [2]. Experts have attempted to develop patient selection criteria for ECMO, but have yet to reach a universal consensus [3]. Universal guidelines have not been established due to factors such as lack of robust research evidence, regional patient heterogeneity, diverse clinical settings, proximity to a large transplant center, resource availability, the diverse and complex pathologies that patients present with, as well as the differentiation of selection criteria required for the ECMO-requiring pathology itself [4–6]. Life support with ECMO often poses considerable risks to patients, such as neurological deficit, stroke, and loss of limbs, up to and including death, which should deter gratuitous use by clinicians [7–10].

According to the Extracorporeal Life Support Organization (ELSO) [11], which contains the largest repository of national and international ECMO data, US adult ECMO survival rates until decannulation or hospital transfer are as follows: VV-ECMO, 59%; VA-ECMO, 46%; and ECPR, 30%. Longer-term data from a single-center study showed that the 5-year survival rates were 33% and 36% for VA ECMO and VV ECMO patients, respectively [12]. The study reported that 29% of surviving VA ECMO patients and 75% of surviving VV ECMO patients had great difficulty with basic daily living activities [12]. Of the patients who survived 5 years post-ECMO, 23% of VA ECMO patients and 58% of VV ECMO patients reported a high post-traumatic stress score [12].

The costs of initiating, maintaining, and discontinuing ECMO are substantial. These include, but are not limited to, the expense of ECMO disposables and consoles; round-the-clock staffing with perfusionists, ECMO specialists, and critical care personnel; patient-related costs, including financial, physical, and psychological burdens arising from ECMO-related complications; and hospital costs associated with operating room (OR) use, cardiac catheterization labs, and extended critical care stay [4, 13].

Current clinical practice involving patient selection criteria and decision-making mechanisms varies by site. It is common for sites to have no formalized criteria for ECMO initiation. Site-specific criteria and decision-making protocols are highly variable and lack standardization [14–16]. Due to significant patient morbidity and mortality, there is a need for streamlined initiation criteria and formalized decision-making mechanisms backed up by research and clinical experience. ECMO data-gathering bodies such as ELSO have published patient selection guidelines for VA ECMO, VV ECMO, and ECPR, but stipulate that the decision is ultimately left to the on-site medical team or attending physician [1, 3, 17]. Some sites have specific criteria for ECMO initiation based on their clinical experience, criteria used by other sites, and academic papers. The decision is often left to the attending physician, who may or may not have experience with ECMO. This can lead to suboptimal decision-making about patient selection and a lack of consensus among the interdisciplinary team.

There has been an increase in research publications on indication-based ECMO initiation criteria, although none have been widely tested or used. Some of these criteria may only be trialed or used at one site. Other researchers have endeavored to model prognostic scores to determine VA ECMO candidacy. With the challenges surrounding patient selection, what selection criteria and decision-making mechanisms should be used to initiate adult VA ECMO?

## Background: ELSO VA ECMO criteria

ELSO’s definition of acceptable patient parameters for VA ECMO consideration and initiation is described in Table 1. The data used by ELSO to generate this list of parameters are from the following studies: SHOCK trial [18] (1999), IABP-SOAP II [19] (2012), EHS-PCI [20] (2012), ESC-HF Guidelines [21] (2016), and KAMIR-NIH [22] (2018) [17]. The SHOCK trial (*n* = 292) and IABP-SOAP II (*n* = 600) are randomized trials. EHS-PCI is a prospective, multi-center observational study (*n* = 336). An expert European task force developed the ESC-HF Guidelines, and KAMIR-NIH represents a study of a Korean multi-center registry of patients with acute myocardial infarction (*n* = 1027) [18–21, 23]. As such, ELSO cardiogenic shock (CS) parameters are taken from a mix of unblinded randomized trials, prospective and retrospective observational studies, and expert opinion.

**Table 1:** ELSO’s definition of cardiogenic shock (CS) suitable for VA ECMO [17].

Systemic systolic pressure < 90 mmHg
Urine output <30 mL/h
Lactate >2 mmol/L
SVO_2_ < 60%
Altered conscious state for 6 h, unresponsive to conventional treatment

ELSO also describes common and emerging uses of VA ECMO for various types of CS, as outlined in Table 2 [17]. These indications have evolved, including patient populations who were seldom cannulated in the past, now being considered for ECMO therapy.

**Table 2:** ELSO common and emerging CS indications for VA ECMO [17].

Common CS indications
– Acute myocardial infarction (AMI)
– Myocarditis
– Cardiac arrest
– Post heart transplant/left ventricular assist device (LVAD)
– Post-cardiotomy
– Hypothermia with cardiac instability

Emerging CS indications

– Acute/massive pulmonary embolism (APE)
– Trauma
– Post-partum acute cardiomyopathy
– Drug intoxication
– Sepsis/sepsis-associated cardiomyopathy
– Back-up support for cardiological interventional procedures
– Arrhythmic storm

ELSO stipulates that VA ECMO merits consideration for refractory CS patients with a reversible or correctable cause after medical treatment (i.e., fluids, inotropes, IABP, etc.) fails [17]. VA ECMO should be initiated before multi-organ failure and after echocardiographic evaluation, with strong consideration given to the patient’s prognosis [17]. Table 3 lists VA ECMO contraindications.

**Table 3:** ELSO VA ECMO contraindications [17].

VA ECMO contraindications
– Cardiac recovery unlikely with ineligibility for LVAD or heart transplant
– Poor life expectancy (multi-organ failure, neoplasms, chemotherapy-induced cardiomyopathy, etc.)
– Severe aortic insufficiency
– Severe vascular disease with extensive peripheral and aortic involvement
– Acute Type A or B aortic dissection with extensive aortic branches (ascending, supra-aortic, and femoral) involvement preoperatively
– Severe neurologic impairment
– Severe immunologic disease with blood and coagulation disorders
– Liver cirrhosis (Child-Pugh class B and C)

Postcardiotomy (PC) ECMO remains a challenge, with in-hospital mortality rates exceeding 60% [24]. There is a lack of robust research on PC ECMO patient selection criteria, with most studies having sample sizes of fewer than 50 patients, despite being the most common indication for VA ECMO [4]. The indication for PC ECMO is persistent CS despite optimal inotropic support post-CPB, with no consensus on exactly when to initiate therapy [4]. The Survival After Venoarterial ECMO (SAVE) score performed well in predicting survival in PC ECMO patients despite not being designed for PC ECMO, since it does not account for physiological alterations in patients post-cardiopulmonary bypass (CPB) [4]. Despite this limitation, it showed moderate discrimination for post PC ECMO survival [4]. The most relevant hospital mortality predictor of patients who require PC ECMO is the number of high-dose (2+) inotropes needed to wean from CPB [25]. Uncontrollable bleeding is the only absolute contraindication to PC ECMO [4]. Despite this, PC ECMO is often initiated in patients with significant bleeding. The EACTS, ELSO, STS, and AATS have released joint recommendations for PC-ECLS initiation (Table 4) [4].

**Table 4:** EACTS, ELSO, STS, and AATS joint recommendations for indications, contraindications, and prognostication of PC-ECLS [4].

Recommendations	Class of recommendation	Level of evidence
Lactate level < 4 mmol/L in patients likely to have myocardial recovery and in the absence of uncontrollable bleeding, not amenable to surgical repair	I	B
If likelihood of myocardial recovery is low, PC ECMO is recommended in patients eligible for LVAD or heart transplant	I	C
Early use of PC ECMO in an IABP patient and optimal medical therapy, with failure to wean from CPB or marginal hemodynamics, is recommended	I	B
Significant comorbidities, advanced age, elevated lactate level, and renal injury are risk factors for death and should be considered pre-ECMO	IIa	B
Preoperative implant of ECLS may be considered in patients in poor hemodynamic or metabolic condition or with structural cardiac anomalies (post-acute MI, VSD, severe lung edema or dysfunction due to underlying cardiac disease), to facilitate perioperative management (bridge to surgery).	IIb	C
The type of ECLS discussed should be based on hemodynamic conditions and patient characteristics	IIa	C

## Methods

For VA ECMO patient factor analysis and prognostic score evaluation, the PubMed database was searched using the term “VA ECMO”, which yielded 26,333 results. When PubMed filters were used, the number of studies that met the inclusion criteria was 1648. The following filters were used: articles published 2005 and later for contemporary studies, adults (19+), humans, English articles, and study types: clinical trial, meta-analysis, multicenter study, observational study, randomized controlled trial, review, and systematic review. Exclusion criteria were pediatric/neonatal patients, animal studies, cardiopulmonary bypass (CPB) articles, VAD-only patients, non-English papers, n < 50, and articles published before 2005.

Inclusion criteria that required additional screening for single-factor analysis papers were: patients undergoing ECMO with VAD, recording and study of pre-ECMO patient selection parameters and associated outcomes, and a sample size of *n* ≥ 50. Additional inclusion criteria assessed for prognostic score papers were scores that had been externally validated with evidence of clinically acceptable discrimination for patient selection (*c*-statistic or AUROC of 0.75 or greater in relevant populations).

**Figure 1:**
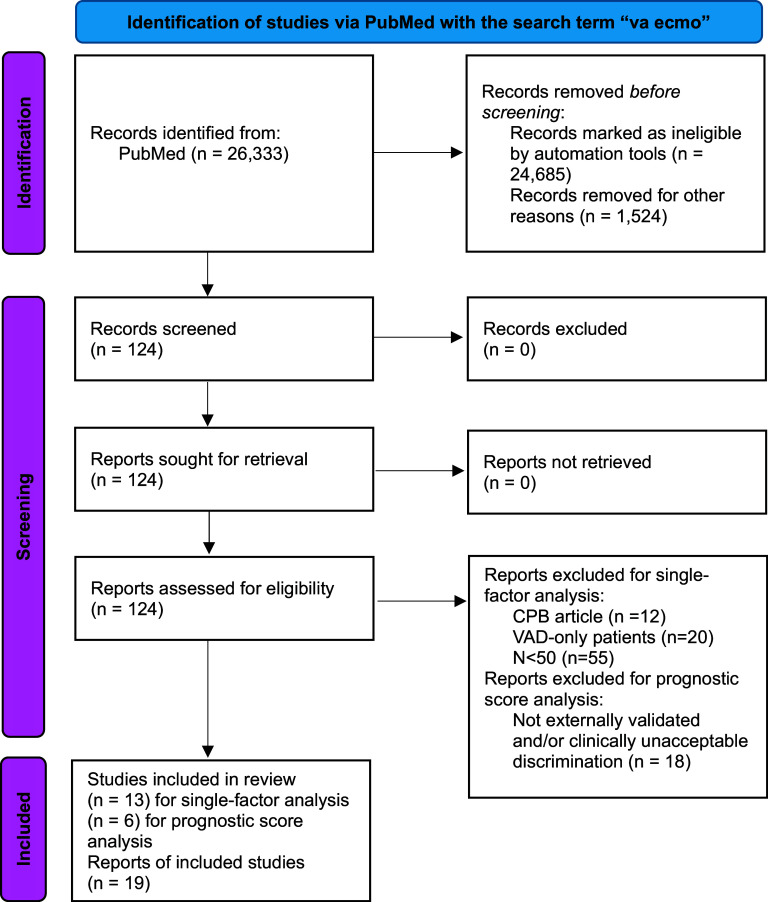
PRISMA diagram.

After screening, 124 papers underwent full-text review. After these articles were reviewed, 13 papers were included for single-factor analysis, and 6 for prognostic score evaluation (see Figure 1) [26–43].

## Significant patient factors pre-VA ECMO

Studies of VA ECMO patient selection parameters often examine a collection of standalone physiologic or clinical patient factors among patients who received ECMO and assess if they are statistically significant in determining patient mortality. The data-extraction summary of the 13 papers analyzed in this section is found in Appendix Tables B, C, and D.

In a single-center retrospective cohort study [31] examining in-hospital cardiac arrest (IHCA) patients, the authors recommended following a decision tree involving initial rhythm, low-flow duration, and age to help identify candidates for IHCA ECMO.

In an IHCA ECMO meta-analysis [32] with 856 patients initial shockable rhythm (49.5% vs. 37.2%, OR 1.65, 95% CI: 1.05–2.61; *p* = 0.03), shorter low-flow time (28.7 ± 4.1 vs. 46.1 ± 5.1 min, *p* < 0.00001), lower pre-ECMO lactate (6.9 ± 0.8 vs. 11.0 ± 0.50 mmol/L, *p* < 0.0001), lower SOFA scores (PMD −1.71 [−2.93, −0.50], *p* = 0.006), and lower pre-ECMO creatinine (1.11 ± 0.05 vs. 1.48 ± 0.06 mg/dL, *p* = 0.003) were found to predict better patient outcomes. Good neurological outcomes, defined as the ability to perform independent activities of daily living at discharge, were predicted by shorter low-flow duration and lower pre-ECMO lactate levels (OR 1.04, 95% CI: 1.00–1.08, and OR 1.31, 95% CI: 1.13–1.52), respectively [32].

In another single-center retrospective cohort study [33] examining PC shock patients, the only two independent risk factors for 90-day mortality after multivariable analysis were the pre-ECMO ischemic heart disease (IHD) and arterial lactate [33]. Pre-ECMO arterial lactate in survivors was between 2.0 and 8.6 (4.0 on average), and between 5.4 and 14.9 (8.0 on average) in non-survivors [33]. Patients with a pre-ECMO arterial lactate of >10 mmol/L had much worse outcomes (*p* < 0.001), and all patients with lactates of 15 mmol/L and greater died within 20 days of ECMO initiation [33]. Ninety-day survival rates differed significantly between patients with and without IHD (23% vs. 69%, *p* < 0.001) [33].

Given low survival rates, PC ECMO in patients over 70 years of age remains controversial and has been examined in an extensive systematic review and meta-analysis by Biancari et al. (*n* = 781) [31]. Upon univariate analysis, arterial lactate >6 mmol/L before initiating VA ECMO predicted hospital mortality, with age > 70 years associated with hospital death (*p* < 0.001), death during VA ECMO (*p* < 0.001), renal replacement therapy (*p* = 0.025), longer ECMO duration (*p* = 0.043), and longer ICU stay (*p* < 0.001) [34].

In a large ELSO registry analysis [35] (*n* = 15,172) of age and VA ECMO outcomes, the authors used the 18–29 year old age group as the reference group and found that adverse age-related outcomes on VA ECMO occur as early as 40 years. The primary diagnoses of these patients before VA ECMO were non-specific cardiogenic shock (29.6%), acute myocardial infarction (7.8%), congestive heart failure (5.6%), and PC ECMO (4.4%) [35].

In the PELS-1 retrospective multi-center study [36] on PC VA ECMO (*n* = 2058), the authors modeled significant patient factors affecting in-hospital mortality at each phase of PC ECMO. In all four models, cardiac arrest and age are associated with in-hospital mortality [36]. Post-discharge mortality is associated with atrial fibrillation, older age (HR: 1.03 [95% CI: 1.02–1.05]), postoperative acute kidney injury (HR: 1.37, [95% CI: 1.01–1.95]), emergency surgery, type of surgery, and septic shock (HR: 2.53, [95%CI: 1.42–4.53]) [36].

A 2024 individual patient data meta-analysis [37] (*n* = 1269) showed that arterial lactate levels at VA ECMO initiation were lower in survivors. The study found that arterial lactate levels before initiation should not exceed 6.8 mmol/L, with in-hospital mortality rates of 76.7% compared to 55.7% (*p* < 0.0001) [37]. The article further details that in-hospital mortality in patients >70 years of age with a pre-ECMO lactate of ≥6.8 mmol/L was 85.2%, and should be considered very conservatively for ECMO treatment [37].

Another retrospective single-center study [38] (*n* = 152) found that the initial pre-ECMO blood lactate level greater than 6.25 mmol/L showed moderate discrimination for mortality (AUROC: 0.731). This study also found that a lower admission sequential organ failure assessment (SOFA) score and the presence of atrial fibrillation had prognostic value for PC ECMO [38].

In a smaller retrospective ELSO registry analysis study [39] (*n* = 96) analyzing calcium channel blocker toxicity patients on VA ECMO, pre-ECMO renal replacement therapy (OR: 10.08, 95% CI: 1.95–52.00, *p* = 0.006) and pre-ECMO acidosis with pH < 7.1 (OR: 3.87, 95% CI: 1.15–12.99, *p* = 0.028) were found to be significantly associated with in-hospital mortality using multivariate analysis.

Another retrospective multivariate analysis of the CS: utility and efficacy of device therapy (RESCUE) registry [40] (*n* = 789) for all VA ECMO indications found that in hospital mortality was predicted by older age (OR: 1.019, *p* = 0.007), chronic liver disease (OR: 8.87, *p* = 0.04), pre-ECMO cardiac arrest (OR: 2.76, *p* = 0.006), elevated total bilirubin (OR: 1.093, *p* < 0.0001) with pre-ECMO sinus rhythm having a protective effect (OR: 0.374, *p* = 0.006). It was also noted that PC ECMO patients had the highest mortality rates (37.2% vs. 28.9%; *p* = 0.02) [40]. Older age was found to be significant for in-hospital mortality, with the average age of survivors being 52.1 years and non-survivors 56.7 years (*p* < 0.0001) [40].

A 2022 systematic review and meta-analysis [41] of 1162 VA ECMO patients with ST-elevated myocardial infarction (STEMI) complicated by CS found that anterior wall infarction (OR: 1.69, 95% CI: 1.37–2.07), longer time from arrest to ECMO (OR: 1.63, 95% CI: 1–2.66), BMI >25 kg/m^2^ (OR: 3.9, 95% CI: 0.92–17.25), lactate >8 mmol/L (OR: 2.90, 95% CI: 0.36–23.61), longer CPR time (OR: 1.85, 95% CI:1.21–2.83), and age > 65 years (OR: 2.50, 95% CI: 1.48–4.24) were predictors of mortality with attainment of TIMI-3 flow after PCI being protective (OR: 0.12, 95% CI: 0.04–0.34).

A single-center retrospective study [42] (*n* = 177) examining 1-year outcomes of non-surgical VA ECMO patients found that only ECPR was independently associated with increased mortality (OR: 3.67, 95% CI: 1.66–8.31, *p* < 0.01).

VA ECMO post-heart transplant for early graft dysfunction (EGD) is becoming more popular [43]. A 2023 systematic review and meta-analysis [43] analysing aggregate and individual patient data for 1477 patients showed that older recipient age (OR: 1.02, 95% CI: 1.01–1.04) and older donor age (OR: 1.01, 95% CI: 100–1.03) slightly increased short-term and 1-year mortality while prior sternotomy had a more significant association with mortality (OR: 1.57, 95% CI: 0.99–2.49) [43].

## Results of single factor analysis

When looking at VA ECMO criteria generalized to all indications, a few parameters appeared recurrently as significant for mortality (see Table B). These parameters were longer low-flow time (significant in 2 papers), higher pre-ECMO lactate (significant in 6 papers), higher SOFA score (significant in 2 papers), higher pre-ECMO creatinine (significant in 3 papers), older age (significant in 7 papers), and pre-ECMO cardiac arrest (significant in 3 papers). The only indication that had any factors repeated at least twice in the studies reviewed was PC ECMO, as it was the most studied VA ECMO indication. The significant pre-ECMO factors for PC ECMO were lower pre-ECMO lactate (significant in 4 papers), lower pre-ECMO creatinine (significant in 2 papers), and older age (significant in 2 papers).

## VA ECMO prognostic score analysis and results

There are at least 16 prognostic scores for VA ECMO patients, of which few have been externally validated [44]. The most common variables included in prognostic scores are age, lactate, creatinine, bilirubin, and the number of days of mechanical ventilation before ECMO, in that order [44]. This paper will focus only on ECMO scores that have been externally validated with clinically acceptable discrimination for VA ECMO selection: the SAVE score, the modified SAVE score, the 6-h PREDICT VA ECMO score, and the 12-h PREDICT VA ECMO score [44].

ELSO briefly mentions VA ECMO prognostic scores, with the greatest coverage of the SAVE score [17]. The SAVE score was developed using ELSO registry data and is utilized for VA ECMO survival prediction, except for PC ECMO and ECPR (see Appendix Table A) [17, 45]. The International Society of Heart and Lung Transplantation (ISHLT) and the Heart Failure Society of America (HFSA) guidelines state that the Simplified Acute Physiology Score (SAPS)-II and the SAVE score are superior to other scores in predicting survival to discharge in VA ECMO patients [46, 47]. The SAVE score has been both internally and externally validated, demonstrating moderate external and internal discrimination for all VA ECMO indications, with near-perfect calibration internally and mild over-prediction of risk externally (Table 5) [26, 45]. According to a retrospective analysis of the ELSO registry [26], the SAVE score shows excellent discrimination with an area under the receiver operating characteristics (AUROC) of 0.90 (95% CI: 0.85–0.95). Another retrospective single-center study [28] analyzing 120 patients found that VA ECMO survivors had a significantly higher SAVE score compared to non-survivors. This study also found that SAVE score risk classes overpredicted mortality, as patients who fit into risk classes II–V exhibited higher survival rates than predicted by the SAVE score (II: 67% vs. 58%, III: 78% vs. 42%, IV: 61% vs. 30%, V: 29% vs. 18%) which could lead to exclusion of patients who would benefit from VA ECMO. The SAVE score demonstrated good discrimination (*c* = 0.77, 95% CI: 0.69–0.86, *p* < 0.001) in this cohort [28, 48]. It is important to note that the SAVE score was not validated in ECPR patients in this study, but did include PC ECMO patients [28]. The SAVE score between ECPR patients who survived vs. those who did not survive did not significantly differ (−10.2 ± 4.0 vs. −12.8 ± 5.2, *p* = 0.198) [28]. In another analysis [27], the discriminatory performance of the SAVE score was found to be better in cohorts without ECPR patients compared to cohorts with ECPR patients (*c* = 0.74, 95% CI: 0.59–0.84; vs. *c* = 0.70, 95% CI: 0.64–0.75). The SAVE score has shown superior predictive power for 30-day mortality of VA ECMO patients (HR: 1.06 (95% CI: 1.03–1.09), *p* < 0.001) as compared to other VA ECMO prognostic scores [26]. It has been externally validated over 20 times [27]. A criticism of the SAVE score is its tendency to overestimate mortality rates, particularly in low to moderate risk classes [27, 28, 49]. SAVE score mortality rates from classes I–V require discrimination testing for diagnostic use.

**Table 5:** Externally validated VA ECMO scores with statistical information.

Score name	Study	Tested patient population	Statistical Info
SAVE score	Schmidt et al. [26] (*n* = 3846)	– Adult VA ECMO patients	*Internal validation*:
			– *c*-statistic (95% CI): 0.68 (0.66–0.69)
			– O:E ratio (95% CI): 0.99
	Pladet et al. [45] (*n* = N/A)	– Adult VA ECMO patients, including ECPR	*External validation (ECPR)*:
			– *c*-statistic (95% CI): 0.70 (0.64–0.75) in pooled analysis
			– O: E ratio (95% CI): 1.15 (0.88–1.49) in pooled analysis
		– Adult VA ECMO patients excluding ECPR	*External validation (no ECPR)*:
			– *c*-statistic (95% CI): 0.74 (0.59–0.84)
			– O:E ratio (95% CI): 1.13 (0.70–1.80)
	Schmidt et al. [26] (*n* = 161)	– Adult VA ECMO patients except PC and ECPR	*External validation*:
			AUROC: 0.90 (95% CI: 0.85–0.95)
	Amin et al. [28] (*n* = 120)	– Adult VA ECMO patients for refractory CS and ECPR	*External validation*:
			– Survivors vs. non-survivors SAVE score:
			−9.3 ± 4.1 vs. −13.1 ± 4.4, *p* = 0.001
			– *c*-statistic (95% CI): 0.77 (0.69–0.86), *p* < 0.001
			– limited predictive value: survival rates by SAVE score class: II: 67% vs. 58%, III: 78% vs. 42%, IV: 61% vs. 30%, V: 29% vs. 18%
Modified SAVE score	Chen et al. [29] (*n* = 154)	– Emergency department adult VA ECMO – includes ECPR, excludes PC ECMO	*External validation*:
			– SAVE +lactate: AUROC: 0.84
	Santore et al. [50] (*n* = 126)	– Adult VA ECMO cardiac arrest patients, CS, PC ECMO	*External validation*:
			– All indications *c*-statistic (95% CI): *c* = 0.736 (0.643–0.829), *p* < 0.001
			– Excluding PC ECMO *c*-statistic (95% CI): *c* = 0.744 (0.645–0.844), *p* < 0.001
PREDICT VA-ECMO (6-h and 12-h)	Wengenmayer et al. [30]	– all adult VA ECMO patients	*External validation*:
			− 6 h of ECMO: AUROC (95% CI): 0.718 (0.65–0.78)
			− 12 h of ECMO: AUROC (95% CI): 0.735 (0.65–0.82)

The modified SAVE score, which includes lactate levels in the SAVE score, has also shown promise for VA ECMO prognostication (see Appendix Table E). In a retrospective cohort study [29] of 154 patients who received VA ECMO in the emergency department (ED), including ECPR, the SAVE score (HR: 0.92, 95% CI: 0.88–0.96, *p* = 0.001) and lactate level (HR: 1.01, 95% CI: 1.01–1.01, *p* < 0.001) were independently associated with VA ECMO patient outcomes. The modified SAVE score demonstrated good discrimination (AUROC 0.84) for predicting outcomes in the ED [29]. A 2021 single-center retrospective study [50] conducted to validate the modified SAVE score (*n* = 126) found that the modified SAVE score showed greater discrimination and outperformed the SAVE score in all VA ECMO indications (*c* = 0.736 (95% CI: 0.643–0.829), *p* < 0.001; vs. *c* = 0.610 (95% CI: 0.509–0.712), *p* = 0.040 respectively). This effect was particularly pronounced in PC and ECPR patients (see Table 5) [50]. Further research is needed to assess the predictive value of the modified SAVE score risk classes, as well as to conduct more extensive external validation of model discrimination.

The PREDICT VA ECMO score uses lactate, pH, and bicarbonate values at 6 h or 12 h into VA ECMO to predict hospital mortality [30]. This criterion originates from a single-center study analyzing 205 VA ECMO patients, with no consideration given to indication, and demonstrates good discrimination (after 6 h: AUROC 0.718; after 12 h: AUROC 0.735) [30]. The PREDICT score is dynamic and can be used for CS and ECPR patients [30]. The PREDICT score showed superior discrimination compared to the SAVE score (AUROC: 0.823 vs. 0.686) in 6-h ECMO survivors [30]. It is essential to note that the PREDICT score is specifically designed to assess the outcomes of patients on ECMO and does not utilize values before ECMO initiation to predict survival, thereby limiting its application in decision-making for ECMO initiation [30].

## ECMO decision-making mechanisms: A multidisciplinary process

The ISHLT and the HFSA advocate for a multidisciplinary team-based initiation approach to maximize patient survival on ECMO [46]. Multidisciplinary evaluation for patient selection by a shock team utilizing algorithms is a class I recommendation for decision-making around the allocation of acute mechanical circulatory support (MCS) [46].

The most notable benefit of an ECMO group is that all members have expertise in ECMO, and all involved disciplines can voice their concerns and contribute to determining solutions before a final decision is made. In the few studies available, formalized multidisciplinary ECLS teams including perfusionists, respiratory therapists, and nurses have been shown to improve survival in retrospective studies [51, 52]. A retrospective study [52] (*n* = 70) compared survival rates of ARDS ECMO patients before and after institution of a formalized ECMO team and found that ICU mortality (72.9 vs. 50.0%, *p* = 0.012) and in-hospital mortality (75.7 vs. 52.2%, *p* = 0.009) were significantly down in the ECMO team era [52]. Significantly more ECMO patients were weaned (42.9 vs. 65.2%, *p* = 0.018) with a substantial decline in 1-year mortality (37.8 vs. 14.3%, *p* = 0.005) [52].

In another retrospective chart review study [51] (*n* = 279), the development of an ECMO team also significantly increased survival to discharge (37.7% vs. 52.3%, *p* = 0.02). In this study, the multidisciplinary ECMO team consisted of perfusionists, intensivists, respiratory therapists, ICU nurses, cardiac surgeons, cardiac anesthesiologists, nutritionists, physical therapists, occupational therapists, a cardiology heart failure specialist, and an ethics committee member [51]. These results are promising and merit further study.

Multidisciplinary consultation before VA ECMO initiation is a formalized process involving all groups directing the patient’s care once they are on ECMO (i.e., intensivists, cardiothoracic surgeons, perfusionists, advanced practice providers (APPs), etc.). A clinical ethicist should also be present to offer their expertise and mediate discussions as needed [46, 53]. All participants can communicate their concerns and have them documented formally. A scenario where this may not be practical is ECMO initiations on cardiac arrest patients, necessitating quick decision-making. It is appropriate to have one ECMO physician make this decision in this instance after consulting the patient selection criteria. In high-risk patients, ECMO eligibility should be pre-emptively discussed before procedures or treatment, allowing for a well-informed decision to be made before a cardiorespiratory event occurs. The decision should be formally documented and put into the patient’s chart under their code status. A streamlined process for VA ECMO decision-making, concentrated in the hands of staff with expertise in ECMO, is needed. This approach presents numerous possibilities for optimizing patient selection for ECMO at the site level.

## Limitations and future directions

The body of research on VA ECMO selection criteria primarily consists of retrospective or prospective observational single-center or multi-center studies, supplemented by some systematic reviews or meta-analyses of these studies. There is a paucity of high-quality, randomized studies with large sample sizes on VA ECMO patient selection criteria, an issue also mentioned in ELSO’s VA ECMO recommendations article and other articles [17, 32]. Studies that analyze patient selection data and scores use data from patients who went on VA ECMO. No studies have compared patients with similar presentations who were not treated with VA ECMO and their outcomes with conventional medical therapy. This is a significant limitation of the research and should be an avenue for future studies to compare outcomes between patients with similar presentations who were treated with VA ECMO versus those who were not. Possible confounding factors, such as pre-ECMO Impella/VAD support or pre-ECMO CRRT affecting ECMO patient outcomes, are minimally discussed. Further research is needed to investigate how these factors impact VA ECMO patient outcomes and whether they should be considered in ECMO patient selection criteria. Independent patient data (IPD) meta-analyses on this topic are absent from the literature, which would allow for adjustment of major confounders such as pre- and post-ECMO usage of left ventricular unloading devices, use of Impella/VAD, or pre-ECMO CRRT usage [32].

There are a few VA ECMO RCTs that investigate patient selection criteria, which have hindered the development of evidence-based recommendations [15]. It is difficult to conduct RCTs for VA ECMO due to the ethics associated with denying a patient life-saving therapy to meet the condition of randomization. RCTs performed on VA ECMO indication-specific patient selection criteria will better elucidate the difference in ECMO selection needed for patients of varying indications who receive ECMO compared to those who do not. Consideration of ethics is essential for VA ECMO RCTs. Because randomization is problematic, the body of evidence is limited to retrospective and prospective studies, prognostic scores modeled after the ECMO registry and retrospective data, and expert opinion [15].

Another significant issue is the lack of standardization in selection criteria studies. Definitions for selection criteria, ECMO complications, protocols for pharmacologic management, use of selection criteria, sample sizes, timing of initiation, indications treated with ECMO, use of an ECMO data registry, data availability from other centers, and mechanisms for decision-making differ significantly by study and center [14–16]. This makes the data produced hard to compare, reproduce, and statistically model in a meaningful way. Standardization of study methodology and definitions is paramount in conducting VA ECMO patient selection studies moving forward to draw meaningful conclusions.

Guidance of VA ECMO patient selection decisions using clinical and research-backed criteria can improve ECMO patient outcomes at sites delivering ECMO therapy. All ECMO centers should adopt patient selection criteria to guide cannulation decisions; it is no longer acceptable to provide this high-risk, life-saving therapy without pre-established criteria to inform the decision-making process.

One retrospective study [36] pointed out that following strict criteria for VA ECMO selection at their site would have resulted in not offering the therapy to 58% of ineligible patients who received ECMO and survived. This problem exists in part because current ECMO eligibility criteria are based on ECMO type (i.e., VA, VV, ECPR), when ECMO patient selection criteria should be personalized based on patient pathology, not ECMO type. Significantly more study is needed on indication-specific ECMO patient selection criteria, which could lead to better ECMO patient outcomes. This is especially critical for special patient groups such as drug overdose patients, post-heart and/or lung transplant patients, patients with neoplasms, pregnant patients, and post-partum patients. Few studies are available for these groups, and the available studies have small sample sizes [39, 43, 54, 55].

## Healthcare implications: Proposed VA ECMO patient selection criteria

Based on existing research, the most promising VA ECMO patient selection criteria for all VA ECMO indications, excluding ECPR and PC ECMO, is the SAVE score. The SAVE score has been rigorously externally validated compared to other VA ECMO scores. Additional research on the discrimination of the SAVE score risk classes in patient cohorts that exclude PC ECMO and ECPR is needed. Most studies tested patient cohorts without either PC ECMO patients or ECPR patients, but not both. A study by Schmidt et al. [26], which excluded PC ECMO and ECPR patients, demonstrated high discrimination of the SAVE score (AUROC: 0.90).

For PC ECMO and ECPR indications, the modified SAVE score has shown good discrimination (ECPR: AUROC 0.84, PC ECMO: *c* = 0.744, *p* < 0.001) [29, 50]. The inclusion of lactate in the selection criteria has value in emergency scenarios such as PC ECMO and ECPR. In PC ECMO, the modified SAVE score has demonstrated superior discrimination compared to the SAVE score (AUROC: 0.695 vs. 0.672) [56]. Further research is needed to externally validate the modified SAVE score in PC ECMO and ECPR patient cohorts, while predictive value should be assessed for each risk class.

## VA ECMO universal criteria: Feasible?

Currently, universal selection criteria have insufficient evidentiary support. The existing research base for ECMO patient selection criteria is burdened with low-level evidence, and indication-specific studies and decision-making mechanism studies are too nascent to produce conclusive directives. Other factors that further complicate standardization include regional patient heterogeneity, diverse clinical settings, proximity to a transplant center, expanding and diverse patient pathologies considered for ECMO, and resource availability. In theory, universal ECMO criteria should improve efficiency of resource use, reduce costs to healthcare systems, and ameliorate most ethical considerations around which patients receive ECMO by introducing standardization. Universal VA ECMO prognostic criteria could be feasible with a stronger body of evidence (i.e., RCTs and meta-analyses of RCTs), greater gathering and sharing of standardized data across centers and healthcare systems nationally, standardization of study format with well-defined selection criteria used, and statistical modeling and external validation of prognostic scores.

## Conclusion

Adult VA ECMO patient selection criteria and the mechanism by which ECMO initiation decisions are made remain the most significant challenge to decreasing patient mortality on VA ECMO. There are substantial weaknesses in the existing research on patient selection criteria, including the use of small sample sizes, a lack of RCTs, a lack of study standardization, a lack of personalization of ECMO initiation decisions based on patient pathology, and conducting studies on patients who received ECMO without including patient groups that were considered for ECMO but did not receive it. Among the available studies, the SAVE score has shown the greatest promise in selecting VA ECMO patients, excluding those undergoing ECPR and PC ECMO. The modified SAVE score has shown the greatest promise in selecting ECPR and PC ECMO patients for VA ECMO. The consultation of a multidisciplinary ECMO team, which collectively makes decisions about ECMO candidacy, has shown significantly increased patient survival outcomes compared to a one- or two-physician decision-making mechanism for VA ECMO initiation. There is currently insufficient support for universal VA ECMO selection criteria, which could be feasible in the future with a stronger body of research evidence.

## Abbreviations

VA ECMO: Venoarterial extracorporeal membrane oxygenation
ECMO: Extracorporeal membrane oxygenation
SAVE score: Survival after venoarterial ECMO score
ECPR: Extracorporeal cardiopulmonary resuscitation
PC: Postcardiotomy
VV: Venovenous
VAD: Ventricular assist device
ELSO: Extracorporeal Life Support Organization
CS: Cardiogenic shock
CPB: Cardiopulmonary bypass
IHCA: In-hospital cardiac arrest
IHD: Ischemic heart disease
SOFA: Sequential Organ Failure Assessment
STEMI: ST-Elevated myocardial infarction
EGD: Early graft dysfunction
ISHLT: International Society of Heart and Lung Transplantation
HFSA: Heart Failure Society of America
SAPS: Simplified Acute Physiology Score

## Data Availability

Data are available in the appendices.

## Appendix

**Table A:** The SAVE score parameters and assigned scores [17].

Parameter		Score
Acute cardiogenic shock diagnosis group (select one or more)		
– Myocarditis		3
– Refractory VT/VF		2
– Post heart or lung transplant		3
– Congenital heart disease		−3
– Other diagnoses leading to cardiogenic shock		0
Age (years)		
– 18–38		7
– 39–52		4
– 53–62		3
– ≥63		0
Weight (kg)		
– ≤65		1
– 65–89		2
– ≥90		0
Acute pre-ECMO organ failures		
– Liver failure (bilirubin ≥33 umol/L or ALT/AST >70 UI/L)		−3
– Central nervous system (CNS) dysfunction (neurotrauma, stroke, encephalopathy, cerebral embolism, seizure/epileptic syndromes)		−3
– Renal failure (acute renal insufficiency: creatinine >1.5 mg/dL with or without RRT)		−3
– Chronic renal failure (kidney damage or GFR <60 mL/min/1.73 m2 for ≥3 mos.)		−6
Duration of intubation before initiation of ECMO (hrs)		
– ≤10		0
– 11–29		−2
– ≥30		−4
Peak inspiratory pressure ≤ 20 cm H_2_O		3
Pre-ECMO cardiac arrest		−2
Diastolic blood pressure pre-ECMO ≥40 mmHg		3
Pulse pressure before ECMO ≤20 mmHg		−2
HCO_3_ before ECMO ≤15 mmol/L		−3
Constant value to add to all calculations of SAVE score		−6
Total score		−35 to 17
Total SAVE score	Risk class	Survival (%)
>5	I	75
1–5	II	58
−4 to 0	III	42
−9 to −5	IV	30
≤−10	V	18

**Table B:** Breakdown of significant pre-VA-ECMO patient factors with associated indications and study details.

Significant pre-ECMO patient factor	Associated VA ECMO indication	Statistical information by study	Study sample size	Study type
Presence of initial shockable rhythm	In-hospital cardiac arrest [28, 29]	– 49.5% vs. 37.2%, OR 1.65, 95% CI: 1.05–2.61; *p* = 0.03	*N* = 856	Systematic review and meta-analysis
		– 53.3% vs. 24.3%, *p* < 0.01	*N* = 137	Single-center retrospective cohort study
Shorter low-flow time (arrest to ECMO time)	In-hospital cardiac arrest [28, 29]	– 28.7 ± 4.1 vs. 46.1 ± 5.1 min, *p* < 0.00001	*N* = 856	Systematic review and meta-analysis
		– 30 (25–53) vs.50 (35–80) min, *p* < 0.01	*N* = 137	Single-center retrospective cohort study
	STEMI with CS [38]	– Arrest to ECMO time: OR: 1.63, 95% CI: 1.00–2.66	*N* = 1162	Systematic review and meta-analysis
		– CPR time: OR: 1.85, 95% CI: 1.21–2.83		
Lower pre-ECMO lactate	In-hospital cardiac arrest [29]	– 6.9 ± 0.8 vs. 11.0 ± 0.50 mmol/L, *p* < 0.0001	*N* = 856	Systematic review and meta-analysis
	Postcardiotomy shock [30]	– OR per unit: 1.22, 95% CI: 1.07–1.40, *p* = 0.004	*N* = 107	Single-center retrospective cohort study
	Postcardiotomy shock >70 yrs [31]	– Pre-ECMO lactate >6 mmol/L (82.6% vs. 70.4%, *p* = 0.029)	*N* = 781	Systematic review and meta-analysis
	Postcardiotomy shock [34]	– Lactate 9.3 mmol/L in non-survivors vs. 6.6 mmol/L in survivors, *p* < 0.0001	*N* = 1269	Individual patient data meta-analysis
	Postcardiotomy shock [35]	– Initial level > 6.25 mmol/L AUROC: 0.731, OR: 1.21, 95% CI: 1.016–1.721, *p* = 0.032	*N* = 152	Retrospective single-center study
	STEMI with CS [38]	– Lactate >8 mmol/L: OR: 2.90, 95% CI: 0.36–23.61	*N* = 1162	Systematic review and meta-analysis
Pre-ECMO acidosis	Calcium channel blocker toxicity [36]	– pH ≥ 7.1 vs. pH < 7.1: OR: 3.87, 95% CI: 1.15–12.99, *p* = 0.028	*N* = 98	Retrospective ELSO registry analysis
Lower SOFA score at ECMO initiation	In-hospital cardiac arrest [29]	– PMD −1.71 [−2.93, −0.50], *p* = 0.006	*N* = 856	Systematic review and meta-analysis
	Postcardiotomy shock [35]	– 13.5 vs. 9, *p* < 0.001	*N* = 152	Retrospective single-center study
Lower pre-ECMO creatinine	In-hospital cardiac arrest [29]	– 1.11 ± 0.05 vs. 1.48 ± 0.06 mg/dL, *p* = 0.003	*N* = 856	Systematic review and meta-analysis
	Postcardiotomy shock [33]	– Survivors: 79.6–128 (avg. 98.1) vs non-survivors: 80–148.5 (avg. 105.6), *p* = 0.003	*N* = 2058	Retrospective international multi-center study
	Postcardiotomy shock [35]	– CKD: 26.9 vs. 6.3%, *p* = 0.004	*N* = 152	Retrospective single-center study
Pre-ECLS renal replacement therapy	Calcium channel blocker toxicity [36]	– OR: 10.08, 95% CI: 1.95–52.00, *p* = 0.006	*N* = 98	Retrospective ELSO registry analysis
Liver disease	All VA ECMO indications [37]	– Chronic liver disease: OR: 8.87, *p* = 0.04 – Elevated total bilirubin: OR: 1.093, *p* < 0.0001	*N* = 789	Registry for CS: utility and efficacy of device therapy retrospective analysis
Subsequent cardiovascular intervention (surgery or PCI)	In-hospital cardiac arrest [28]	– 63.3% vs. 26.2%, *p* < 0.01	*N* = 137	Single-center retrospective cohort study
BMI	STEMI with CS [38]	– BMI >25 kg/m^2^: OR: 3.98, 95% CI: 0.92–17.25	*N* = 1162	Systematic review and meta-analysis
Older age	In-hospital cardiac arrest [28]	– Age ≥ 55 years, multivariable OR: 4.8 95% CI: [1.7–15.4], *p* = 0.005	*N* = 137	Single-center retrospective cohort study
	Postcardiotomy shock >70 yrs [31]	– Age > 70 years (76.1% vs. 58.7%, adjusted OR: 2.199, 95% CI: 1.536–3.149)	*N* = 781	Systematic review and meta-analysis
	Postcardiotomy shock [33]	– Non-survivors average age 67 (58–73) vs. survivors average age 61.75 (52.2–70), *p* < 0.001	*N* = 2058	Retrospective international multi-center study
	Cardiogenic shock [32]	– 40–49 (OR: 1.26, [95% CI:1.08–1.47]) – 50–59 (OR:1.78, [95% CI: 1.55–2.06]) – 60–69 (OR: 2.24 [95% CI: 1.94–2.59]) – 70–79 (OR: 2.90 [95% CI: 3.13–5.20]) – OR: 1.019, *p* = 0.007	*N* = 15,172	ELSO registry retrospective analysis
	All VA ECMO indications [37]	– Age > 65 years: OR: 2.50, 95% CI: 1.48–4.24	*N* = 789	Registry for CS: utility and efficacy of device therapy retrospective analysis
	STEMI with CS [38]	– Per year of age: OR:	*N* = 1162	Systematic review and meta-analysis
	Early graft dysfunction post heart transplant [40]	– 1.02, 95% CI: 1.01–1.04	*N* = 1477	Systematic review and meta-analysis
Other mechanical support	In-hospital cardiac arrest [28]	– Univariable OR: 0.3 95%CI: [0.1–0.9], *p* = 0.03	*N* = 137	Single-center retrospective cohort study
Ischemic heart disease	Postcardiotomy shock [30]	– OR: 7.87, 95% CI: 2.55–24.3, *p* < 0.001	*N* = 105	Single-center retrospective cohort study
	STEMI with CS [38]	– Anterior wall infarction: OR: 1.69, 95% CI: 1.37–2.07	*N* = 1162	Systematic review and meta-analysis
Pre-operative cardiac arrest	Postcardiotomy shock [33]	– HR: 1.34, 95% CI: 1.08–1.66, *p* = 0.0073	*N* = 2058	Retrospective international multi-center study
Pre-ECLS cardiac arrest	All VA ECMO indications [37]	– OR: 2.76, *p* = 0.006	*N* = 789	Registry for CS: utility and efficacy of device therapy retrospective analysis
	All VA ECMO indications [39]	– OR: 3.67, 95% CI: 1.66–8.31, *p* < 0.01	*N* = 177	Retrospective single-center study
Pre-operative cardiogenic shock	Postcardiotomy shock [33]	– 17.9% in survivors vs. 23.6% in non-survivors, *p* = 0.002	*N* = 2058	Retrospective international multi-center study
Pre-operative septic shock	Postcardiotomy shock [33]	– 1.3% in survivors vs. 3.3% in non-survivors, *p* = 0.005	*N* = 2058	Retrospective international multi-center study
Pre-operative cardiac diagnosis	Postcardiotomy shock [33]	– Aortic vessel disease: 13.4% in survivors vs. 18.2% in non-survivors, *p* = 0.003 – Aortic valve disease: 27.8% in survivors vs. 38.2% in non-survivors, *p* < 0.001 – Tricuspid valve disease: 13.9% in survivors vs. 17.4% in non-survivors, *p* = 0.032	*N* = 2058	Retrospective international multi-center study
Atrial fibrillation	Postcardiotomy shock [35]	– Non-survivors 41.35% vs. 12.5% in survivors, *p* < 0.001	*N* = 152	Retrospective single-center study
Prolonged CPB time	Postcardiotomy shock [35]	– 237 min vs. 192 min, *p* = 0.016	*N* = 152	Retrospective single-center study
Aortic cross-clamp times	Postcardiotomy shock [35]	– 160 vs. 124 min, *p* = 0.04	*N* = 152	Retrospective single-center study
Prior sternotomy	Early graft dysfunction post heart transplant [40]	– OR: 1.57, 95% CI: 0.99–2.49	*N* = 1477	Systematic review and meta-analysis
Older donor age	Early graft dysfunction post heart transplant [40]	– OR: 1.01, 95% CI: 1.00–1.03	*N* = 1477	Systematic review and meta-analysis

**Table C:** Breakdown of significant and insignificant pre-VA-ECMO patient factors by indication.

Indication	Study	Study type	Insignificant patient factors	Significant patient factors
In-hospital cardiac arrest	Arrigo et al. [32]	Systematic review and meta-analysis	– Age – Gender – Cardiac vs. non-cardiac etiology	– Initial shockable rhythm – shorter low flow time – lower pre-ECMO lactate – lower SOFA score – lower pre-ECMO creatinine
In-hospital cardiac arrest	Bourcier et al. [31]	Single-center retrospective cohort study	– Age (in isolation) – Cardiac arrest cause – Cardiac arrest location – BMI – Sex	– Initial shockable rhythm – Shorter low-flow time – Subsequent cardiovascular intervention – Age (multivariate analysis with low-flow time and presence of shockable rhythm) – Other mechanical support
Postcardiotomy shock	Fux et al. [33]	Single-center retrospective cohort study	– Pre-ECMO hemodialysis – Pre-ECMO CPR and IABP – CPB time – Cross-clamp time – Redo surgery – BMI – Sex – Atrial fibrillation	Multivariate analysis: – Pre-ECMO lactate – Ischemic heart disease Univariate analysis: – Age – Pre-ECMO LVEF – Pre-ECMO MAP
Postcardiotomy shock age > 70 yrs	Biancari et al. [34]	Systematic review and meta-analysis	– PRBC transfusion – Redo surgery – Arterial complications – Liver failure	– Age – Pre-ECMO lactate
Postcardiotomy shock	Mariani et al. [36]	Retrospective international multi-center study	– Sex – BMI – CAD – Pulmonary valve disease – Endocarditis – ASD – Preoperative vasopressors	In-hospital mortality: – Age – Preoperative cardiogenic shock – Preoperative cardiac arrest – Preoperative septic shock – Preoperative cardiac diagnosis (aortic and valvular) – Preoperative creatinine
Postcardiotomy shock	Biancari et al. [37]	Individual patient data meta-analysis	– Central cannulation vs. peripheral	– Higher pre-ECMO lactate level – Pre-ECMO lactate level + age
Postcardiotomy shock	Laimoud et al. [38]	Retrospective single-center study	– Acute ischemic stroke – Cerebral bleeding – Bowel surgery	– Higher pre-ECMO lactate – Atrial fibrillation – CKD – Higher CPB time – Higher aortic cross-clamp time – Lower SOFA score – Lower SAVE score
Cardiogenic Shock	Fernando et al. [35]	ELSO registry retrospective analysis	– Age 30–39	– Age as early as 40 years associated with greater mortality
Calcium channel blocker toxicity	Subramanian et al. [39]	ELSO registry retrospective analysis	– Pre-ECLS cardiac arrest – Arrhythmia – Sepsis – Neurologic complications – Age – BMI	– Pre-ECLS renal replacement therapy – Pre-ECLS severe acidosis (pH < 7.1)
All VA ECMO indications	Vigneshwar et al. [40]	Registry for CS: utility and efficacy of device therapy retrospective analysis	– Prior PCI – BMI – Postcardiotomy shock – Lactate pre-ECMO – Hypertension – Diabetes – ACS – Stroke	– Older age – Pre-ECLS cardiac arrest – Chronic liver disease – Elevated total bilirubin – Pre-ECMO sinus rhythm was protective
All VA ECMO indications	Ingvarsdottir et al. [42]	Retrospective single-center study	– Underlying etiology – pH – Lactate – Biventricular failure	– ECPR
STEMI with CS	Pang et al. [41]	Systematic review and meta-analysis	N/A	– TIMI-3 flow after PCI (protective) – BMI > 25 kg/m^2^ – Arrest to ECMO time – CPR time – Lactate >8 mmol/L – Anterior wall infarction – Age > 65 years
Early graft dysfunction post-heart transplant	Aleksova et al. [43]	Systematic review and meta-analysis	– Recipient sex – Donor sex – Ischemic time – Pretransplant VAD	– Older recipient age – Prior sternotomy – Increasing donor age

**Table D:** Breakdown of significant pre-VA-ECMO patient factors by study, and measured outcomes.

Study	Significant patient factors	Relevant outcomes of study
Arrigo et al. [32]	– Initial shockable rhythm: 49.5% vs. 37.2%, OR 1.65, 95% CI: 1.05–2.61; *p* = 0.03 – Shorter low-flow time: 28.7 ± 4.1 vs. 46.1 ± 5.1 min, *p* < 0.00001 – Lower pre-ECMO lactate levels: 6.9 ± 0.8 vs. 11.0 ± 0.50 mmol/L, *p* < 0.0001 – Lower SOFA score (PMD –1.71 [−2.93, −0.50], *p* = 0.006) – Lower pre-ECMO creatinine: 1.11 ± 0.05 vs. 1.48 ± 0.06 mg/dL, *p* = 0.003 – Shorter low-flow time and lower blood lactate during CCPR associated with CPC 1–2 (OR 1.04 [1.00–1.08] and 1.31 [1.13–1.52], respectively)	– Primary: survival to discharge (IHCA patients) – Secondary: rate of favorable neurological outcome at discharge (cerebral performance categories (CPC) 1 or 2 – absent to moderate disability)
Bourcier et al. [31]	– Initial shockable rhythm (53.3% vs. 24.3%, *p* < 0.01) – Shorter low-flow time (30 (25–53) vs.50 (35–80) min, *p* < 0.01) – Subsequent cardiovascular intervention (63.3% vs. 26.2%, *p* < 0.01) – Age ≥ 55 (multivariate analysis with low-flow time and presence of shockable rhythm) (multivariable OR: 4.8, 95% CI: [1.7–15.4], *p* = 0.005) – Other mechanical support (univariable OR: 0.3 95%CI: [0.1–0.9], *p* = 0.03)	– Primary: in ICU mortality – Secondary: 1-year survival post-hospital discharge with good neurological outcome
Fux et al. [33]	– Pre-ECMO lactate (OR per unit: 1.22, 95% CI: 1.07–14.0, *p* = 0.004) – Ischemic heart disease (OR: 7.87, 95% CI: 2.55–24.3, *p* < 0.001)	– Association between ECMO implant variables and all-cause mortality at 90 days
Biancari et al. [34]	– Pre-ECMO lactate >6 mmol/L (82.6% vs. 70.4%, *p* = 0.029) – Age > 70 years (76.1% vs. 58.7%, adjusted OR: 2.199, 95% CI: 1.536–3.149)	– In-hospital mortality, or within 30 days after cardiac surgery
Mariani et al. [36]	– Age: non-survivors average age 67 (58–73) vs. survivors average age 61.75 (52.2–70), *p* < 0.001 – Pre-operative cardiac arrest HR: 1.34, 95% CI: 1.08–1.66, *p* = 0.0073 – Pre-operative cardiogenic shock 17.9% in survivors vs. 23.6% in non-survivors, *p* = 0.002	– In-hospital mortality – Post-discharge mortality
Biancari et al. [37]	– Pre-ECMO lactate level: lactate 9.3 mmol/L in non-survivors vs. 6.6 mmol/L in survivors, *p* < 0.0001 – Pre-ECMO lactate level + age (see text)	– In-hospital mortality – On VA ECMO mortality
Laimoud et al. [38]	– Pre-ECMO lactate: initial level > 6.25 mmol/L AUROC: 0.731, OR: 1.21, 95% CI: 1.016–1.721, *p* = 0.032 – Atrial fibrillation: Non-survivors 41.35% vs. 12.5% in survivors, *p* < 0.001 – CKD: CKD: 26.9 vs. 6.3%, *p* = 0.004 – CPB time: 237 min vs. 192 min, *p* = 0.016 – Aortic cross-clamp time: 160 vs. 124 min, *p* = 0.04 – SOFA score: 13.5 vs. 9, *p* < 0.001 – SAVE score: −3 vs. 3, *p* < 0.001	– In-hospital mortality
Fernando et al. [35]	– Age at ECMO initiation: 40–49 (OR: 1.26, [95% CI:1.08–1.47]) 50–59 (OR:1.78, [95% CI: 1.55–2.06]) 60–69 (OR: 2.24 [95% CI: 1.94–2.59]) 70–79 (OR: 2.90 [95% CI: 3.13–5.20])	– In-hospital mortality
Subramanian et al. [39]	– Pre-ECLS renal replacement therapy: OR: 10.08, 95% CI: 1.95–52.00, *p* = 0.006 – Pre-ECLS pH ≥7.1 vs <7.1: OR: 3.87, 95% CI: 1.15–12.99, *p* = 0.028	– In-hospital mortality
Vigneshwar et al. [40]	– Age: OR: 1.02, 95% CI: 1.008–1.032, *p* = 0.0008 – Total bilirubin pre-ECMO OR: 1.082, 95% CI: 1.082–1.017, *p* = 0.01 – History of chronic liver disease: OR: 9.486, 95% CI: 1.0165–77.26, *p* = 0.04 – Pre-ECMO sinus rhythm (protective): OR: 0.39, 95% CI: 0.222–0.685, *p* = 0.001 – Pre-ECMO cardiac arrest: OR: 1.735, 95% CI: 1.084–2.775, *p* = 0.02	– In-hospital mortality
Pang et al. [41]	– TIMI-3 flow after PCI (protective): OR: 0.12, 95%CI: 0.04–0.34 – BMI >25 kg/m^2^: OR: 3.98, 95% CI: 0.92–17.25 – Arrest to ECMO time: OR: 1.63, 95% CI: 1–2.66 – CPR time: OR: 1.85, 95% CI: 1.21–2.83 – Lactate >8 mmol/L: OR: 2.90, 95% CI: 0.36–23.61 – Anterior wall infarction: OR: 1.69, 95% CI: 1.37–2.07 – Age > 65 years: OR: 2.50, 95% CI: 1.48–4.24	– Predictors of short-term mortality – Incidence of complications during VA ECMO run
Aleksova et al. [43]	Short term mortality: – Advancing recipient age: OR: 1.02, 95% CI: 1.01–1.04 – Prior sternotomy: OR: 1.57, 95% CI: 0.99–2.49 – Older donor age: OR: 1.01, 95% CI: 1.00–1.03 1-year mortality: – Older recipient age: OR: 1.02, 95% CI: 1.00–1.04 – Prior sternotomy: OR: 1.56, 95% CI: 1.56, 95% CI: 1.00–2.43 – Older donor age: OR: 1.01, 95% CI: 1.00–1.03	– Mortality on VA ECMO – Short-term mortality (30 day or in-hospital) − 1-year mortality
Ingvarsdottir et al. [42]	– ECPR: OR: 3.67, 95% CI: 1.66–8.31, *p* < 0.01	− 1-year outcomes post- ECMO

**Table E:** The Modified SAVE score parameters and assigned scores [29].

Parameter		Score
Acute cardiogenic shock diagnosis group (select one or more) – Myocarditis – Refractory VT/VF – Post heart or lung transplant – Congenital heart disease – Other diagnoses leading to cardiogenic shock		3 2 3 −3 0
Age (years) – 18–38 – 39–52 – 53–62 – ≥63		7 4 3 0
Weight (kg) – ≤65 – 65–89 – ≥90		1 2 0
Acute pre-ECMO organ failures – Liver failure (bilirubin ≥33 umol/L or ALT/AST >70 UI/L) Central nervous system (CNS) dysfunction (neurotrauma, stroke, encephalopathy, cerebral embolism, seizure/epileptic syndromes) – Renal failure (acute renal insufficiency: creatinine >1.5 mg/dL with or without RRT) – Chronic renal failure (kidney damage or GFR <60 mL/min/1.73 m^2^ for ≥3 mos.)		−3 −3 −3 −6
Duration of intubation before initiation of ECMO (hrs) – ≤10 – 11–29 – ≥30		0 −2 −4
Peak inspiratory pressure ≤ 20 cm H_2_O		3
Pre-ECMO cardiac arrest		−2
Diastolic blood pressure pre-ECMO ≥40 mmHg		3
Pulse pressure before ECMO ≤20 mmHg		−2
HCO_3_ before ECMO ≤15 mmol/L		−3
Constant value to add to all calculations of SAVE score		−6
Lactate (mmol/dL) – <7.5 – ≥7.5		15 0
Total score		−35 to 32
Total SAVE score	Risk class	Survival (%)
>10	I	79
1–10	II	52
−4 to 0	III	32
−10 to −5	IV	10
≤−10	V	4
